# A cross-sectional nationwide survey of congenital and infantile nephrotic syndrome in Japan

**DOI:** 10.1186/s12882-020-02010-5

**Published:** 2020-08-24

**Authors:** Yuko Hamasaki, Riku Hamada, Masaki Muramatsu, Shinsuke Matsumoto, Kunihiko Aya, Kenji Ishikura, Tetsuji Kaneko, Kazumoto Iijima

**Affiliations:** 1 Department of Nephrology, Toho University Faculty of Medicine, 6-11-1 Omori-Nishi, Ota-Ku, Tokyo, 143-8541 Japan; 2 Department of Nephrology, Tokyo Metropolitan Children’s Medical Center, 2-8-29 Musashidai, Fuchu, Tokyo, 183-8561 Japan; 3 Department of Pediatrics, Matsudo City General Hospital, 993-1, Sendabori, Matsudo, Chiba, 270-2296 Japan; 4 Department of Pediatrics, Kurashiki Central Hospital, 1-1-1 Miwa, Kurashiki, Okayama, 710-8602 Japan; 5 Department of Pediatrics, Kitasato University School of Medicine, 1-15-1 Kitazato, Minami-Ku, Sagamihara, Kanagawa 252-0375 Japan; 6 Division of Clinical Research Support Center, Tokyo Metropolitan Children’s Medical Center, 2-8-29 Musashidai, Fuchu, Tokyo, 183-8561 Japan; 7 Teikyo Academic Research Center, Teikyo University, 2-11-1 Kaga, Itabashi-Ku, Tokyo, 173-8606 Japan; 8 Department of Pediatrics, Kobe University Graduate School of Medicine, 7-5-2 Kusunoki-cho, Chuo-Ku, Kobe, Hyogo 650-0017 Japan

**Keywords:** Congenital nephrotic syndrome, Complete remission, Extra-renal symptoms, End-stage kidney disease, Finnish-type disease, Infantile nephrotic syndrome, Japan, Survey

## Abstract

**Background:**

Congenital nephrotic syndrome (CNS) and infantile nephrotic syndrome (INS) cause substantial morbidity and mortality. In Japan, there is a lack of knowledge regarding the characteristics of CNS and INS. This study aimed to clarify the characteristics of CNS and INS in Japan.

**Methods:**

This cross-sectional nationwide survey obtained data from 44 institutions in Japan managing 92 patients with CNS or INS, by means of two survey questionnaires sent by postal mail. Patients aged < 16 years by 1 April 2015, with a diagnosis of CNS or INS, were included in this study. The primary outcome was end-stage kidney disease.

**Results:**

A total of 83 patients with CNS or INS were analyzed. The most frequent disease type was non-Finnish (60.2%); 33 patients (39.8%) had Finnish type. Among those with non-Finnish-type disease, 26 had no syndrome and 24 had a syndrome, of which the most frequent was Denys–Drash syndrome (70.8%). Patients with non-Finnish-type disease with syndrome showed the earliest progression to end-stage kidney disease compared with the other two groups, whereas patients with non-Finnish-type disease without syndrome progressed more slowly compared with the other two groups. In the Finnish-type group, the disease was diagnosed the earliest; a large placenta was reported more frequently; genetic testing was more frequently performed (93.8%); mental retardation was the most frequent extra-renal symptom (21.2%); and thrombosis and infection were more frequent compared with the other groups. Patients with non-Finnish-type disease with syndrome had a higher frequency of positive extra-renal symptoms (79.2%), the most common being urogenital symptoms (54.2%). Treatment with steroids and immunosuppressants was more frequent among patients with non-Finnish-type disease without syndrome. Two patients with non-Finnish-type disease without syndrome achieved complete remission. In all groups, unilateral nephrectomy was performed more often than bilateral nephrectomy and peritoneal dialysis was the most common renal replacement therapy.

**Conclusions:**

The present epidemiological survey sheds light on the characteristics of children with CNS and INS in Japan. A high proportion of patients underwent genetic examination, and patient management was in accord with current treatment recommendations and practices.

**Trial registration:**

Not applicable.

## Background

Congenital nephrotic syndrome (CNS) occurs within 3 months of birth and infantile nephrotic syndrome (INS) onset occurs from 4 to 12 months after birth [1]. Both syndromes cause substantial morbidity. According to the Japanese clinical practice guideline for nephrotic syndrome, nephrotic syndrome in children is defined as the presence of massive proteinuria (≥40 mg/h/m^2^) plus hypoalbuminemia (serum albumin ≤2.5 g/dL) [2]. CNS and INS are also characterized by edema, hyperlipidemia, and hypercoagulable states [3, 4]. CNS is associated with various syndromes, such as Denys–Drash syndrome, Galloway–Mowat syndrome, Pierson syndrome, and nail–patella syndrome [4]. CNS is often resistant to therapy with corticosteroids and immunosuppressive drugs because its pathogenesis is non-immunological. Therefore, management of CNS includes the control of edema, prevention of complications such as infection and thrombosis, and proper nutrition; however, in most cases kidney transplantation is ultimately required [4]. For INS, management generally includes combined treatment with steroids and immunosuppressants [2].

One of the main causes of CNS is Finnish-type disease [5], which belongs to a group of rare hereditary disorders overrepresented in the Finnish population and reported much less frequently in various ethnic groups worldwide [6]. In Finnish-type CNS, some patients may present with irregular pseudocystic dilatation of the proximal tubules [6]; however, there is no specific pathologic characteristic that defines this rare disease. Most children with Finnish-type disease are born prematurely and have a placental weight > 25% of the newborn weight [1]. For patients with Finnish-type disease, treatment with early bilateral nephrectomy and initiation of dialysis have been reported to be effective, followed by renal →kidney transplantation [7]. Other reports have concluded that unilateral nephrectomy is an effective alternative to bilateral nephrectomy for patients with NS [8, 9].

There is currently a lack of knowledge regarding the characteristics of CNS and INS in Asia, specifically in Japan. Therefore, the present observational study aimed to clarify the characteristics of CNS and INS in Japan.

## Methods

### Study design

A cross-sectional nationwide survey was conducted in Japan. A survey was sent by postal mail to 1860 hospitals (with ≥20 beds and a pediatrics department) on 1 April 2015 to request information on which hospitals were treating children with CNS or INS (< 16 years old). The hospitals were selected because children with apparent CNS or INS were usually referred to institutions meeting the above criteria. All surveys returned by 31 May 2015 were evaluated. The response rate for the survey was 63.3%, and among the institutions that responded, 50 institutions managing 130 children with CNS or INS were identified.

A second survey requesting data for individual patients, including age, sex, presence of a syndrome (Denys–Drash syndrome, Galloway–Mowat syndrome, Pierson syndrome, nail–patella syndrome, or other), perinatal characteristics, renal pathology, whether genetic testing was conducted, extra-renal symptoms, treatment, and complications, was sent in February 2016 to the 50 identified institutions. A sample of the survey questionnaire is provided in Additional file 1. All surveys returned by 31 May 2016 were evaluated.

The present study was conducted in accordance with the ethical principles set out in the Declaration of Helsinki and the ethical guidelines for epidemiological studies issued by the Ministry of Health, Labour and Welfare in Japan. The study was approved by the ethics committee of Toho University Omori Medical Center (ID:26–236 for the first survey, ID:27–181 for the second survey). As data were obtained from patient medical records, informed consent was waived. The data in the records were de-identified for protection of the patients’ personal and health information.

### Study subjects

Patients aged < 16 years by 1 April 2015, with a diagnosis of CNS or INS, were included in this study. Nephrotic syndrome was defined as having a urine protein/creatinine ratio ≥ 2.0 and serum albumin ≤2.5 g/dL. CNS was defined as onset of NS within 3 months of birth. INS was defined as onset of NS from 4 to 12 months after birth. There were no exclusion criteria.

### Statistical analysis

Categorical variables (survey items) were summarized by frequency and percentages. Continuous variables were summarized by median and range. The primary outcome was end-stage kidney disease, defined as starting dialysis or requiring preemptive transplantation. The diagnosis date was used as the starting point for survival analysis. Time to end-stage kidney disease and the cumulative proportion of progression were estimated by Kaplan–Meier analysis.

The number of patients with CNS in Japan was estimated from the reported number of patients in our survey. We conducted a comprehensive survey of all facilities where the target cases may continue outpatient visits. Because response rates tend to be lower in institutions with fewer patients, simple estimates may overestimate the true prevalence. Therefore, the reported patients were stratified according to institution type (i.e., university hospital, children’s hospital or general hospital) and the number of beds (< 200, 200–500 or > 500), based on the assumption that the response rate is independent of the number of patients in each stratified category [10]. Then, the number of reported patients in each category was divided by the response rate and summed to calculate the total estimated number of patients in Japan. The total estimated number of patients was divided by the size of the population at risk in Japan reported by the Statistics Bureau of Japan to calculate the prevalence as of 1 April 2015 [11].

Missing data were handled using the missing completely at random mechanism or pair-wise case deletion. All statistical analyses were carried out using SAS system version 9 (SAS Institute, Inc., Cary, NC, USA).

## Results

Of the 50 institutions that were sent the second survey, 44 institutions managing 92 patients with CNS or INS responded. Nine patients who did not meet the diagnostic criteria for CNS or INS were excluded; therefore, a total of 83 patients were included in the present study. Among Japanese children aged 0–15 years, the estimated prevalence of CNS was 0.76 cases/100,000 children, and that of Finnish-type CNS was 0.30 cases/100,000 children. There were 0.24 cases/100,000 children with non-Finnish-type without syndrome and 0.22 cases/100,000 children with non-Finnish-type with syndrome.

The baseline demographic and clinical characteristics of the patients are shown in Table 1. Thirty-eight of the 83 patients (45.8%) were male. The disease type was Finnish in 33 patients (39.8%) and non-Finnish in 50 patients (60.2%). Among those with non-Finnish-type disease, 26 had no syndrome and 24 had a syndrome (Denys–Drash syndrome, Galloway–Mowat syndrome, Pierson syndrome, nail–patella syndrome, or other). Fifty-nine of the 83 patients had no syndrome, and among them, the disease type was Finnish in 33 patients (55.9%) and non-Finnish in 26 patients (44.1%). Among the 24 patients with a syndrome, the most frequent syndrome was Denys–Drash syndrome in 17 patients (70.8%).

**Table 1:** Baseline demographic and clinical characteristics

	Total ***N*** = 83	Finnish-type disease ***N*** = 33	Non-Finnish-type without syndrome ***N*** = 26	Non-Finnish-type with syndrome ***N*** = 24
Male	38	15	14	9
Female	45	18	12	15
Without syndrome	59	33	26	0
With syndrome	24	0	0	24
DDS	17	0	0	17
GM	0	0	0	0
Pierson	4	0	0	4
NPS	0	0	0	0
Other	0	0	0	0

*DDS* Denys–Drash syndrome, *GM* Galloway–Mowat syndrome, *NPS* nail–patella syndrome

The perinatal characteristics of the patients are shown in Table 2. Finnish-type disease was diagnosed the earliest (median age at diagnosis 0.0 months, range 0.0–2.0 months) compared with the other two groups. A large placenta was reported more frequently among patients with Finnish-type disease compared with those with non-Finnish-type disease (with and without syndrome).

**Table 2:** Perinatal characteristics

	Finnish-type disease N = 33	Non-Finnish-type without syndrome N = 26	Non-Finnish-type with syndrome N = 24
Age at diagnosis, months, median (range)	0.0 (0.0–2.0)	7.0 (0.0–12.0)	1.0 (0.0–10.0)
Gestational week (range)	36w4d (30w6d–40w6d)	39w5d (35w4d–42w0d)	38w4d (33w3d–41w3d)
Height at birth, cm, median (range)	45.6 (32.4–50.0)	48.0 (45.8–54.0)	47.3 (42.5–51.0)
Weight at birth, g, median (range)	2298 (1061–3066)	3078 (2036–3580)	2759 (1493–3600)
Oligohydramnios (+)^a^	6/22	1/17	1/14
Large placenta (+)^a^	30/30	4/17	4/12

*d* days, *w* weeks

^a^The denominator represents the number of patients for whom responses regarding oligohydramnios and large placenta were obtained

The results of renal pathology, genetic testing, and extra-renal symptoms are shown in Table 3. Renal biopsy was more frequently performed among patients with non-Finnish-type disease (without syndrome: 23/26, 88.5%; with syndrome: 22/22, 100%) compared with those with Finnish-type disease (13/30, 43.3%). Genetic testing was more frequently performed among patients with Finnish-type disease (30/32, 93.8%) and those with non-Finnish-type with syndrome (23/24, 95.8%) compared with those with non-Finnish-type without syndrome (18/26, 69.2%). Positive extra-renal symptoms were found in 12 of 33 (36.5%) patients with Finnish-type disease and 19 of 24 (79.2%) patients with non-Finnish-type disease with syndrome. The most common extra-renal symptoms were mental retardation (*n* = 7, 21.2%) and other (*n* = 6, 18.2%) among patients with Finnish-type disease; other (*n* = 3, 11.5%) and mental retardation (*n* = 2, 7.6%) among those with non-Finnish-type disease without syndrome; and urogenital (*n* = 13, 54.2%), mental retardation (n = 7, 29.2%), and other (n = 7, 29.2%) among those with Finnish-type disease with syndrome.

**Table 3:** Renal pathology, genetic testing, and extra-renal symptoms

	Finnish-type disease N = 33	Non-Finnish-type without syndrome N = 26	Non-Finnish-type with syndrome ***N*** = 24
Renal biopsy^a^	13/30	23/26	22/22
Genetic testing^a^	30/32	18/26	23/24
Positive extra-renal symptoms^a^	12/33	5/26	19/24
Eyes	1	0	4
Urogenital	0	1	13
Malformation	1	1	1
Epilepsy	4	1	3
Mental retardation	7	2	7
Other^b^	6	3	7

^a^The denominator represents the number of patients for whom responses regarding renal biopsy, genetic testing, and positive extra-renal symptoms were obtained

^b^Other symptoms included a variety of symptoms not necessarily related to congenital nephrotic syndrome

Patients’ treatment and complications are shown in Table 4. Treatment with steroids and immunosuppressants was more frequent among patients with non-Finnish-type disease without syndrome. Among these patients, complete remission was achieved in three patients treated with steroids and in nine patients treated with immunosuppressants. Cyclosporine was the most frequently prescribed immunosuppressant. In the other two groups, none of the patients achieved complete remission regardless of treatment. Infection was frequent in patients with Finnish-type disease frequent in patients with Finnish-type disease.

**Table 4:** Treatments and complications

	Finnish-type disease N = 33	Non-Finnish-type without syndrome N = 26	Non-Finnish-type with syndrome N = 24
Medications^a^			
Steroids	3/29 (NR, 3)	18/25 (NR, 11; PR, 4; CR, 3)	8/24 (NR, 7; PR, 1)
Immunosuppressants	3/28 (CSA, NR, 2; PR, 1)	12/25 (NR, 1; PR, 2; CR, 9)	4/24 (NR, 4)
Immunosuppressant therapy by response	CSA, NR CSA + MZB, NR CSA, PR	CSA + TAC + MZB, NR CSA, PR (*n* = 2) CSA, CR (*n* = 4) MZB, CR CSA + MZB, CR CSA + CP, CR CSA + MMF + RTX, CR CSA + MZB + RTX, CR	CSA, NR (*n* = 4)
Complications^a^			
Thrombosis	5/32	1/26	1/24
Infections	19/32	7/26	9/24
Other	8/32	9/26	11/24

*Abbreviations: CP* cyclophosphamide, *CR* complete remission, *CSA* cyclosporine, *MMF* mycophenolate mofetil, *MZB* mizoribine, *NR* no response, *PR* partial remission, *RTX* rituximab, *TAC* tacrolimus

^a^The denominator represents the number of patients for whom responses regarding medications and complications were obtained

The characteristics of the nephrectomy and renal replacement therapies are shown in Table 5. In general, unilateral nephrectomy was performed more often than bilateral nephrectomy. Peritoneal dialysis was the most common renal replacement therapy in all groups. The median age at renal→kidney transplantation was the highest at 59 months in patients with Finnish-type disease. All patients who underwent renal→kidney transplantation received a primary graft, and none of these patients required subsequent dialysis therapy or a secondary transplant. One patient with Finnish-type disease (age 5 years) developed nephrotic syndrome post-transplant. The patient was treated with steroids and cyclophosphamide and thereafter achieved complete remission.

**Table 5:** Nephrectomy and renal replacement therapies

	Finnish type disease N = 33	Non-Finnish-type without syndrome N = 26	Non-Finnish-type with syndrome N = 24
Nephrectomy			
Unilateral	25/33	3/26	8/24
Median age during procedure, months (range)	13 (3–96)	9, 26, 36	9 (5–44)
Bilateral	0/33	1/26	8/24
Median age during procedure, months (range)	–	20	21 (7–69)
Renal replacement therapies			
Peritoneal dialysis	26/33	9/26	22/24
Median age during procedure, months (range)	21 (4–90)	9 (0–26)	6 (0–25)
Hemodialysis (with catheter)	1/33	1/26	3/24
Median age during procedure, months (range)	67	3	2, 6, 34
Hemodialysis (with fistula)	0/33	0/26	0/24
Median age during procedure, months (range)	–	–	–
renal → kidney transplantation	17/33	5/26	17/24
Median age during procedure, months (range)	59 (34–96)	26 (20–36)	47 (22–73)

**Fig. 1:**
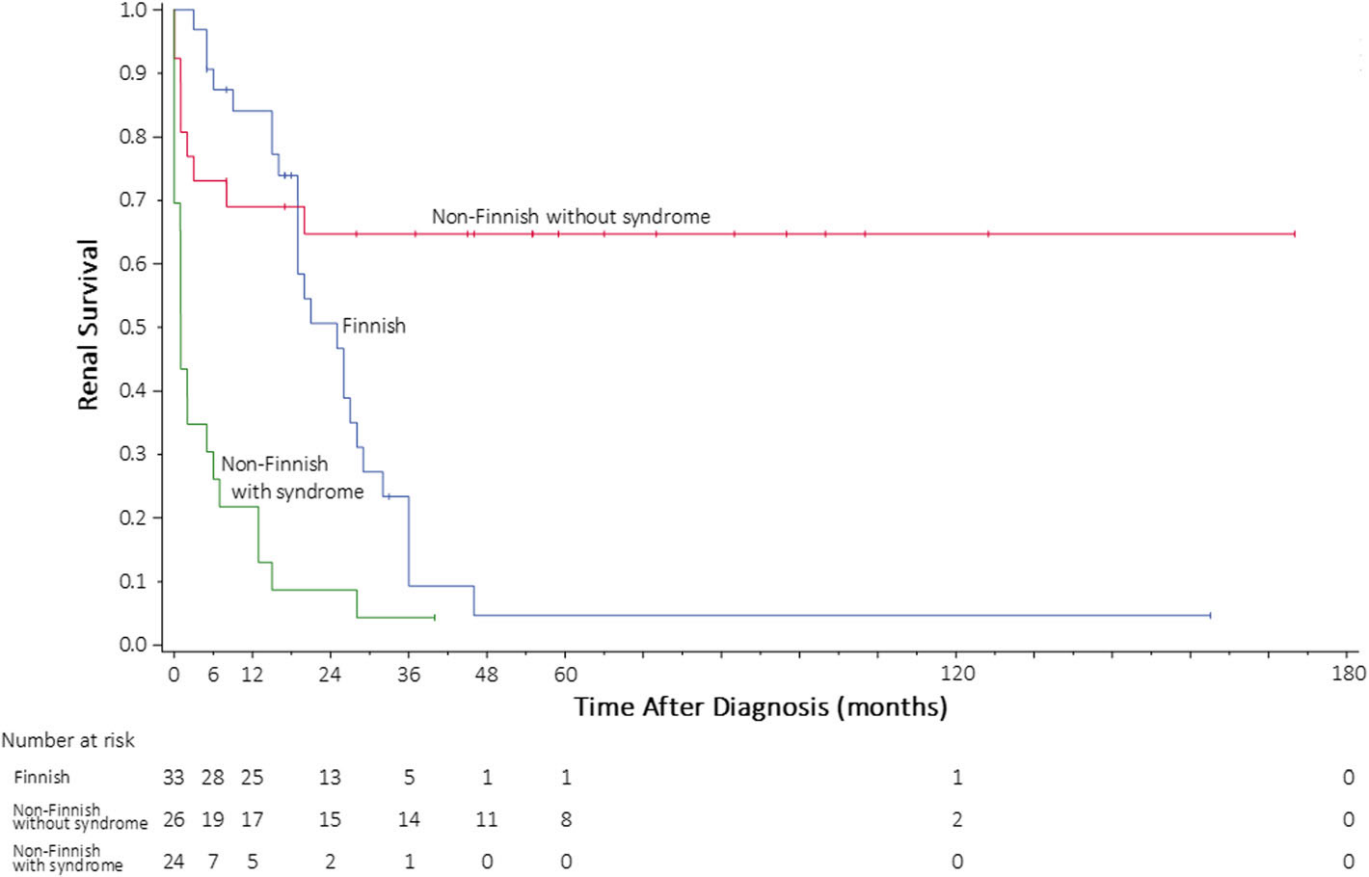
Kaplan–Meier estimate for time to end-stage kidney disease. Blue: Finnish; Red: non-Finnish without syndrome; Green: non-Finnish with syndrome

The Kaplan–Meier analysis for time to end-stage kidney disease is shown in Fig. 1. Patients with non-Finnish-type disease with syndrome showed the earliest progression to end-stage kidney disease compared with the other two groups. Conversely, patients with non-Finnish-type disease without syndrome progressed more slowly compared with the other two groups.

## Discussion

This epidemiological study is the first survey of its kind to investigate the characteristics of CNS and INS in Japan. Data obtained from 44 institutions in Japan managing 92 patients with CNS or INS were evaluated. As a result, we identified and analyzed a total of 83 patients with CNS or INS. In the present study, the estimated prevalence of CNS among Japanese children aged 0–15 years was 0.76 cases/100,000 children, which is comparable to the estimated cumulative incidence of CNS reported in a study of 55 children with CNS in France (0.5/100,000 live births) [12].

In the present study, the disease type was Finnish in 33 patients, non-Finnish without syndrome in 26 patients, and non-Finnish with syndrome in 24 patients. Among patients with Finnish-type disease, the number of male and female patients was similar (15 and 18, respectively). As Finnish-type CNS is inherited in an autosomal recessive manner, the incidence in both sexes tends to be similar [13].

The most frequent syndrome was Denys–Drash syndrome in 70.8% of patients (17/24) in the present study. Denys–Drash syndrome is characterized by nephrotic syndrome owing to diffuse mesangial sclerosis, male pseudohermaphroditism, and predisposition to develop Wilms tumor and gonadoblastoma [14].

Patients with Finnish-type disease were diagnosed the earliest compared with those with non-Finnish-type disease. It is likely that these patients were diagnosed first because of the clinical manifestations of the disease, such as the enlarged placenta, and the massive edema that becomes evident shortly after birth in most patients with Finnish-type CNS. In fact, an enlarged placenta was frequently reported among patients with Finnish-type disease in the present study. This is consistent with the previous descriptions of patients with Finnish-type disease, in which the weight of the placenta is approximately 25% of the birth weight [15].

Renal biopsy was more frequently performed among patients with non-Finnish-type disease, while genetic testing was more frequent among patients with Finnish-type disease and non-Finnish-type with syndrome. Finnish-type CNS does not have characteristic pathologic findings and the diagnosis is usually based on the clinical manifestations along with the results of genetic testing. Several genes (e.g., *NPHS1, NPHS2*, *PLCE1,* and *WT1)* involved in the etiology of CNS and INS have been discovered so far [16–20], and these are useful in the diagnosis of Finnish-type CNS and non-Finnish-type with syndrome [19]. Finnish-type CNS is often associated with mutations in *NPHS1* [19], whereas mutations in *NPHS2* are linked to non-Finnish steroid-resistant INS [20]. In 20% of cases of non-Finnish CNS, Machuca et al. were not able to identify the underlying genetic alteration [20]. Of note, most patients included in this study underwent genetic testing (Finnish type, 30/32 [94%]; non-Finnish type without syndrome, 18/26 [69%]; and non-Finnish type with syndrome, 23/24 [96%]). This is in contrast with a similar study conducted in India, where genetic test results were only available for 15 of 65 (23%) children evaluated [21]. The reasons for this discrepancy may be related to access to laboratories capable of performing such testing. In Japan, such testing—although not covered by national insurance—is readily available.

In the present survey, positive extra-renal symptoms were reported in 36.4, 19.2, and 79.2% of patients with Finnish-type disease and non-Finnish-type without and with syndrome, respectively. Mental retardation (21.2%) was the most frequent extra-renal symptom in patients with Finnish-type disease; in patients with non-Finnish-type disease with syndrome, urogenital symptoms (54.2%) and mental retardation (29.2%) were the most frequent extra-renal symptoms. The presence of urogenital symptoms in patients with non-Finnish-type disease with syndrome may be a useful characteristic for the diagnosis of Denys–Drash syndrome. In a study that evaluated 1655 patients with steroid-resistant nephrotic syndrome from 21 countries [22], extra-renal symptoms were reported in 17.3% of patients, with short stature (*n* = 84, 5.1%) and mental retardation (*n* = 65, 3.9%) being the most common extra-renal symptoms. A higher incidence of extra-renal symptoms was found in the present study compared with the previous study; however, this may be because of the methodological differences between the studies.

In the present study, steroids and immunosuppressants were used more frequently in patients with non-Finnish-type disease without syndrome compared with patients with Finnish-type disease and non-Finnish-type disease with syndrome, and complete remission was only reported among patients with non-Finnish-type disease without syndrome. Generally, patients with Finnish-type and non-Finnish-type with syndrome do not respond to steroids or immunosuppressants. The pathogenesis of Finnish-type CNS is non-immunological; thus, patients with this disease tend to be resistant to treatment with corticosteroids and immunosuppressive drugs [4]. In contrast, patients with non-Finnish-type disease without syndrome are among the INS cases that respond to steroids or immunosuppressants. Interestingly, the present results indicate that most pediatric nephrologists in Japan are aware of such treatment outcomes, and that the patients in this sample were treated in accordance with the recommendations of the Japanese clinical practice guidelines for nephrotic syndrome [2].

Thrombosis and infection are known to be common complications in CNS patients [23]. A single-center study conducted in Jordan reported 25 episodes of serious bacterial infections in 18 out of 30 infants with CNS, most of whom had Finnish-type disease [24]. A retrospective study of 21 infants with Finnish-type CNS conducted in Finland reported 63 verified and 62 suspected episodes of sepsis [25]. In the present study, thrombosis and infection were reported in all groups, but the frequency was particularly high in patients with Finnish-type disease. The high frequency of infection among patients with Finnish-type disease in the present study is consistent with that reported previously in the Jordanian and Finnish studies [24, 25].

The number of patients who underwent unilateral nephrectomy was higher than that of patients who underwent bilateral nephrectomy. The most common renal replacement therapy in all groups was peritoneal dialysis. Although there is currently no guideline for the management of CNS in Japan, unilateral nephrectomy is a more popular approach than bilateral nephrectomy. The main reason for this preference is that the management of patients after unilateral nephrectomy is easier than that after bilateral nephrectomy because dialysis for patients with anuria is more difficult. In a recent retrospective study conducted in Japan, the long-term outcome data from 14 Finnish-type CNS Japanese patients who underwent kidney transplantation showed satisfactory graft survival [26].

The Kaplan–Meier analysis in the present study showed a slower progression to end-stage kidney disease in patients with non-Finnish-type disease without syndrome. The reason may be that this group was the most responsive to treatment, considering it was the only group that included patients who achieved complete remission in response to steroids or immunosuppressive agents.

The present study has some limitations, including those inherent to the observational/cross-sectional study design and those related to the use of questionnaires (incomplete/incorrect completion of survey forms). The response rate of the survey was 63.3%, which may have introduced bias or affected the accuracy of our estimates. Because informed consent from individual patients was not obtained, we could not describe details of specific genetic mutations, or certain details on treatments/medications prescribed. A more detailed investigation of such items is warranted in future studies. Finally, this study focused on Japanese patients, and as such may not be generalizable to other populations.

In conclusion, the present survey sheds light on the characteristics of children with CNS and INS in Japan. Among the most relevant findings, we observed that children with Finnish-type disease were diagnosed earlier than those with non-Finnish-type disease; the placenta was large mainly among those with Finnish-type disease; in patients with non-Finnish-type disease with syndrome, urogenital symptoms were the most frequent extra-renal symptoms; a high proportion of patients underwent genetic examination; and most patients underwent unilateral nephrectomy followed by peritoneal dialysis before renal transplantation. Ideally, all patients with CNS or INS should undergo genetic testing, which may allow a better evaluation of the distribution of these diseases and a better characterization of patients for early stage disease management. In the future, we plan to evaluate the long-term outcomes of CNS and INS.

## Supplementary information

**Additional file 1.** Survey questionnaire.

## Data Availability

The datasets used and/or analyzed during the current study are available from the corresponding author on reasonable request.

## Abbreviations

CNS: Congenital nephrotic syndrome
CP: cyclophosphamide
CR: complete remission
CSA: cyclosporine
DDS: Denys–Drash syndrome
GM: Galloway–Mowat syndrome
INS: infantile nephrotic syndrome
NPS: nail–patella syndrome
MMF: mycophenolate mofetil
MZB: mizoribine
NR: no response
PR: partial remission
RTX: rituximab
TAC: tacrolimus
