# Supplementary material for: A cross-sectional nationwide survey of congenital and infantile nephrotic syndrome in Japan
Source: BMC Nephrol. 2020 Aug 24;21:363. doi: 10.1186/s12882-020-02010-5 (PMC7446144; doi:10.1186/s12882-020-02010-5)
Supplement: Supplementary file 1 — Additional file 1. Survey questionnaire. [file 12882_2020_2010_MOESM1_ESM.docx]

**Congenital or infantile nephrotic syndrome survey**

Birthday (year/month):

Sex:

Diagnosis: □ Finnish type; □ Non-Finnish type

Syndrome: □ Without syndrome

□ Denys-Drash; □ Galloway-Mowat; □ Pierson; □ Nail-patella，

□ Others

Other symptoms not in the kidneys?

　□ Present; □ Absent

□ Eye:

□ Urogenital (Wilms tumor)

□ Malformation:

□ Epilepsy:

□ Mental and/or motor retardation:

□ Others:

Gestational week: 　　 week　 day

Height at birth　　 cm; Weight at birth　　　　g

Oligohydramnios: □ Present　 □ Absent □ Unknown

Large placenta: □ Present (　　g) □ Absent □ Unknown

Family history: □ Present (　 ) □ Absent □ Unknown

Consanguineous marriage: □ Present　　 □ Absent　 □ Unknown

Size of fontanel: at birth　 　×　　 cm □ Unknown

at 3 months　 　×　　 cm □ Unknown

Age when fontanel closed:　　 month(s) □ Unknown

At Onset of Birth

　　 year 　 month (　years　months old)

Height　　 cm; Weight　　　g

Serum creatinine: mg/dL

Serum albumin: 　　 g/dL

Serum IgG: 　　 mg/dL

Proteinuria: mg/dL (– / ± / + / 1+ / 2+ / 3+)

Urine creatinine: 　　mg/dL

6 months after birth

　 months

Height　　 cm; Weight　　　 g

Serum creatinine: mg/dL

Serum albumin: 　　 g/dL

Proteinuria: mg/dL (– / ± / + / 1+ / 2+ / 3+)

Urine creatinine: 　　mg/dL

12 months after birth

　 months

Height　　 cm; Weight　　　 g

Serum creatinine: mg/dL

Serum albumin: 　　 g/dL

Proteinuria: mg/dL (– / ± / + / 1+ / 2+ / 3+)

Urine creatinine: 　　 mg/dL

Latest

　　 year 　 month

Height　　 cm; Weight　　　 kg

Serum creatinine: mg/dL

Serum albumin: 　　 g/dL

Proteinuria: mg/dL (– / ± / + / 1+ / 2+ / 3+)

Urine creatinine: 　　 mg/dL

State of latest:

(normal kidney function, pre-renal replacement therapy, dialysis

[hemo+peritoneal], after kidney transplant, re-dialysis [hemo+peritoneal])

Complications (Thrombosis, infection, or other):

Kidney biopsy: □ Performed　　 □ Not performed

Results of the kidney biopsy:

Genetic test: □ Performed　　 □ Not performed

**Medical therapy**

Steroid therapy:

□ Performed　　 □ Not performed

Response to steroid therapy:

□ Complete remission　 □ Partial remission　　□ No response

Immunosuppressant therapy:

□ Performed (name: 　　　　　　　)　□ Not performed

Response to immunosuppressant therapy:

□ Complete remission　　□ Partial remission　　□ No response

**Nephrectomy**

Unilateral:

□ Performed (Date: 　　year month; Age: years months)

□ Not performed

Opposite side:

□ Performed (Date: 　　year month; Age: years months)

□ Not performed

Bilateral at the same time:

□ Performed (Date: 　　year month; Age: years months)

□ Not performed

**Dialysis therapy**

Peritoneal dialysis:

□ Performed (Date: 　　year month; Age: years months)

□ Not performed

Hemodialysis (catheter):

□ Performed (Date: 　　year month; Age: years months)

□ Not performed

Hemodialysis (with fistula):

□ Performed (Date: 　　year month; Age: years months)

□ Not performed

**Kidney transplant**

Kidney transplant:

□ Performed (Date: 　　year month; Age: years months)

□ Not performed

Peritoneal dialysis after kidney transplant:

□ Yes (Start date:　　year month; Age: years months)

□ No

Hemodialysis after kidney transplant:

□ Yes (Start date:　　year month; Age: years months)

□ No

Onset of nephrotic syndrome after kidney transplant:

□ Yes (Onset:　　year month; Age: years months)

□ No

If yes,

Treatment:

　　Response to treatment:

□ Complete remission　 □ Partial remission　　□ No response
